# Visual statistical learning in preverbal infants at a higher likelihood of autism and its association with later social communication skills

**DOI:** 10.1371/journal.pone.0300274

**Published:** 2024-05-15

**Authors:** Roberta Bettoni, Chiara Cantiani, Elena Maria Riboldi, Massimo Molteni, Hermann Bulf, Valentina Riva

**Affiliations:** 1 Department of Psychology, University of Milano-Bicocca, Milan, Italy; 2 Scientific Institute, IRCCS E. Medea, Child Psychopathology Unit, Bosisio Parini, Lecco, Italy; Fondazione Policlinico Universitario Gemelli IRCCS, ITALY

## Abstract

Visual statistical Learning (SL) allows infants to extract the statistical relationships embedded in a sequence of elements. SL plays a crucial role in language and communication competencies and has been found to be impacted in Autism Spectrum Disorder (ASD). This study aims to investigate visual SL in infants at higher likelihood of developing ASD (HL-ASD) and its predictive value on autistic-related traits at 24–36 months. At 6 months of age, SL was tested using a visual habituation task in HL-ASD and neurotypical (NT) infants. All infants were habituated to a visual sequence of shapes containing statistically predictable patterns. In the test phase, infants viewed the statistically structured, familiar sequence in alternation with a novel sequence that did not contain any statistical information. HL-ASD infants were then evaluated at 24–36 months to investigate the associations between visual SL and ASD-related traits. Our results showed that NT infants were able to learn the statistical structure embedded in the visual sequences, while HL-ASD infants showed different learning patterns. A regression analysis revealed that SL ability in 6-month-old HL-ASD infants was related to social communication and interaction abilities at 24–36 months of age. These findings indicate that early differences in learning visual statistical patterns might contribute to later social communication skills.

## Introduction

Autism spectrum disorder (ASD) is characterized by deficits in social interaction and communication and by the presence of restricted and repetitive patterns of behaviors [1]. A recent review [2] confirmed that the prevalence of autism spectrum disorder (ASD) has shown a marked increase in the last decades and demonstrated a median prevalence of 65/10,000. Autistic characteristics occur early in life, with an 82% stability of ASD diagnosis by the age of 2 years [3]. However, diagnosis is typically not made before the age of 3–4 years, and the detection of early biomarkers, arising before apparent behavioral and clinical features, represents a significant challenge and an important step to early support, taking advantage of early brain plasticity.

The infant sibling approach offers important advantages in terms of the identification of early biomarkers and their cascading effects on later development. Infant siblings of autistic children are typically referred to as having a higher likelihood of developing ASD (HL-ASD, [4]). A previous study demonstrated that 18.7% of HL-ASD infants would themselves receive a diagnosis of ASD at 3 years old [5]. Thus, longitudinal studies on the HL-ASD population are well suited to track different developmental trajectories [5–7]. Considerable progress has been made in characterizing behavioral and neurocognitive markers of later ASD, including implementing prospective and longitudinal designs and experimental techniques in assessing developmental pathways to the emergence of autistic features.

Studies on the role of Statistical Learning (SL) skills in the early phases of life have become important in examining the roots of ASD. SL is an implicit learning mechanism that allows individuals to identify statistical regularities embedded in the environment by uncovering the probabilistic information within an auditory, visual, or tactile sequence of elements and thus generating predictive expectations about what pieces of information will come next [8, 9]. In a seminal study, Kirkham et al. [10] habituated 2-, 5-, and 8-month-old infants to a stream of visual shapes organized in pairs defined by transitional probabilities. Specifically, infants viewed 3 pairs of shapes presented in random order, hence, the predictability between shapes within each pair was 100%, whereas the predictability between shapes across pairs was 33%. After being habituated, infants were presented with the familiar sequence and a novel (random) sequence in alternation. All groups of infants looked more at the novel stimuli than at the familiar ones, suggesting that infants were sensitive to statistical regularities by 2 months of age and visual SL remained stable across the first years of life. By using a similar shape-sequence paradigm, Bulf et al. [11] found that newborn infants were able to detect statistical relationships when sequences were composed by four (but not by six) shapes, suggesting that visual SL is functioning since birth. This early sensitivity to the input’s statistical property has been found to be relevant for both language acquisition and social communication [12–18]. For example, infants are able to use statistical information to discover the units embedded in a sequence of dynamic human actions [12] or emotional facial expressions [19], and can use statistical regularities within social action sequences to predict a future action before it begins [e.g., 20] and to discover cues related to a social event structure, such as the agent’s intentions [21, 22].

According to the neuroconstructivist approach [23], early difficulties in SL abilities are thought to have a cascading effect on the emergence of cognitive and social communication delays in the second postnatal year of life [17]. Since our social world is characterized by temporal, spatial, and contextual statistical regularities (e.g., from learning associations between words and their appropriate meanings to social turn-taking in a conversation), early differences in the SL ability might negatively impact the ability to understand patterns in social interactions, including how to respond to social events and/or how to adjust one’s behavior accordingly. Specifically, the prediction coding account in ASD posits that many characteristics of ASD, such as differences in understanding goal-directed behaviors, can potentially be explained by challenges in predictive learning [e.g., 24]. Differences in predictive learning refer to the struggles in pairing antecedents with their resulting consequences, which can be partially explained by inaccuracy in estimating the conditional probabilities governing how the events evolve over time. Given that SL is crucial in making predictions on the environmental sequence of events [16, 25, 26], and prediction abilities among autistic individuals have been shown to be different [24–26], it is important to investigate how SL operates in the HL-ASD population [17].

Several studies have reported variabilities in SL skills in ASD. In an EEG study, Jeste et al. [27], tested 2- to 6-year-old children with a neurotypical development (NT) and with a diagnosis of ASD. Using an oddball paradigm adapted from Kirkham et al. [10], children were familiarized first to a sequence of visual shapes containing 100% of probabilities within pairs and 33% across pairs. Successively, children viewed a sequence of shapes similar to those seen during familiarization, in which 10% of the trials were characterized by a shape followed by an unmatching shape (unexpected trial) for which predictability was equal to 0. In contrast to the control group, the ASD group showed different neural responses related to visual SL skills defined as N1 and P300 components. Additionally, ASD individuals who exhibited a clear neural response to the statistical information, similarly to the NT group, have higher cognitive skills and adaptive social functioning, further supporting the idea that SL in ASD maps onto non-verbal cognition and social skills acquisition. In line with this finding, Scott-Van Zeeland et al. [28] presented ASD school-aged children with sequences of syllables containing statistical and random relationships. The study found no evidence of learning-related changes in brain activity in response to statistical vs. random sequences in ASD children and significant associations between neural correlates of SL and low communication competencies in the ASD sample. Different patterns in processing statistical relationships in ASD are further confirmed by a recent behavioral study in which 2- to 8-year-old children were asked to identify a target image that might be predicted by a cue image 75% (high frequency) or 25% (low frequency) of the time. Results showed that, differently from the NT group, ASD children were not able to use statistical information to make event predictions. Notably, a subgroup of children with ASD showed behavioral patterns similar to NT children, and such variability in SL skills was related to core ASD symptoms [29]. Together, these studies suggested that SL is related to social functioning in autistic individuals and highlighted that the learning ability in children with ASD is characterized by a high heterogeneity.

These findings are supported by recent infant longitudinal studies in which SL abilities have been assessed in the prodromal stage before a diagnosis of ASD. For example, Bettoni et al. [30] found an association between infants’ visual SL and their parents’ autistic traits. Using a visual habituation task, the results showed that infants whose parents showed lower autistic traits were able to catch the statistical structure embedded in a visual sequence, while infants whose parents showed higher autistic traits were not. These results suggest that early dysfunctions in SL might contribute to individual differences in ASD symptoms and to the Broader Autism Phenotype [31]. A recent EEG study [32] on visual SL showed greater neural activation in response to low than high predictable events in 3-month-old HL-ASD siblings, while the reverse patterns were found in a control group of NT infants. Moreover, these different brain responses were linked to cognitive symptoms at 18 months [32]. These results further suggest the presence of distinct learning processes in HL-ASD compared to NT infants, which might act as a precursor for later delays in nonverbal cognitive skills. Even if HL-ASD and NT infants have different learning patterns at the neural level, it is still unknown whether a difference may emerge at the behavioral level [33].

The aims of the present study are (1) to compare early visual SL skills in 6-month-old HL-ASD infants with those of NT infants at the behavioral level and (2) to assess the longitudinal associations between early SL skills at 6 months and later ASD-related traits at 24–36 months (measured by Autism Diagnostic Observation Schedule). Using a well-established method, HL-ASD and NT infants were first habituated to a string of visual shapes organized in three pairs presented in random order, in which the transitional probability between shapes was higher within the pair (1.00) than between pairs (0.33). In the post-habituation test phase, infants were tested for their ability to discriminate between the familiar sequence presented during the habituation and a new one in which the shape was presented in random order [30]. To the best of our knowledge, the present study is the first attempt to examine SL skills at the behavioral level in HL-ASD infants and to investigate their predictive value on ASD-related traits at 24–36 months, a period when children show more stable behavioral symptoms of a potential diagnosis of ASD [34].

On the one hand, we expect NT infants to be able to discriminate between familiar and novel structures, as indexed by looking longer at the novel sequence relative to the familiar one. On the other hand, according to previous infant studies [30, 32], we expect that, as a group, HL-ASD infants would show differences in discriminating between the novel or familiar test stimuli relative to NT. Further, based on the idea that HL-ASD might be characterized by high heterogeneity in their visual SL performance, we expect to find an association between variabilities in SL skills and later social communication outcomes.

## Method

### Participants

The sample was composed of 21 NT and 20 HL-ASD infants who took part in the study. The NT group consists of infants recruited by local advertisement from two hospitals in Northern Italy for participation in a larger ongoing longitudinal study [35, 36], whereas the HL-ASD group was recruited at the IRCCS Medea Institute within the collaboration with the Italian Network for Early Detection of Autism Spectrum Disorders (the NIDA Network) [37, 38]. The criterion for being included in the HL-ASD group was having at least a biological sibling with a certified diagnosis of ASD. Infants were included in the study if: (1) their gestational age was ≥ 36 weeks, (2) their birth weight was ≥ 2500 grams, (3) their Bayley Cognitive Score was ≥ 7 [39] and/or their Griffiths general quotient ≥ 85 [40], (4) they had complete and usable visual SL data.

Data from 3 infants were excluded from the final sample because they did not complete the task (2 NT) or looking time was less than 1 second in one of the test phase trials (1 HL-ASD). The final sample was thus composed of 19 NT (11 boys; age range = 6 months and 13 days—7 months and 19 days) and 19 HL-ASD infants (10 boys; age range = 5 months and 24 days—7 months and 2 days). The two groups were matched for sex (NT: 11 boys and 8 girls; HL-ASD: 10 boys and 9 girls; *χ2* = .11; *p* = .74). Moreover, all infants underwent a cognitive evaluation before the experimental task. Since Griffiths Developmental Scale [40] or Bayley scales [39] were administered, we computed IQ-transformed scores (mean of 100 and SD of 15) for both scales to compare cognitive levels. Descriptive statistics of demographic characteristics are shown in Table 1. No differences in age, gestational weeks, birth weight, socioeconomic status (SES), and cognitive skills have been found between the two groups.

**Table 1:** Descriptive statistics (mean and standard deviation) on individual, demographic, and clinical characteristics for the NT and HL-ASD infants.

	NT (n = 19) *Mean (SD)*	HL-ASD (n = 19) *Mean (SD)*	t-test p-value
Age (months)	6.85 (.28)	6.67 (.30)	.06
Gestational age (weeks)	39.13 (1.59)	39.05 (1.55)	.89
Birthweight (grams)	3352.19 (498.07)	3191.84 (562.67)	.38
SES^†^	62.50 (11.91)	54.00 (19.31)	.12
Cognitive score	106.31 (5.49)	105.15 (8.26)	.61

Note. ^†^SES = socioeconomic status; SES was scored according to the Hollingshead 9-point scale, whereby a score ranging from 10–90 was assigned to each parental job, and the higher of two scores was used when both parents were employed [41].

Written informed consent was obtained from all parents before starting the testing session. The experiment was approved by the Medea Ethical and Scientific Committee.

### Visual SL task

Infants were tested in a sound isolated and dark cabin while seated on their parent’s laps at approximately 60 cm from the stimulus presentation monitor. Stimuli were generated using E-prime 2.0 software and presented on a 21-inch monitor with 1280 x 720 pixels resolution. A video camera above the monitor recorded the infant’s face and sent a visual input to another computer monitor, thus allowing the online coding of infants’ looking times by an experimenter blind to the stimuli presented. The image of the infant’s face was also video recorded for the offline coding of looking times.

**Fig 1:**
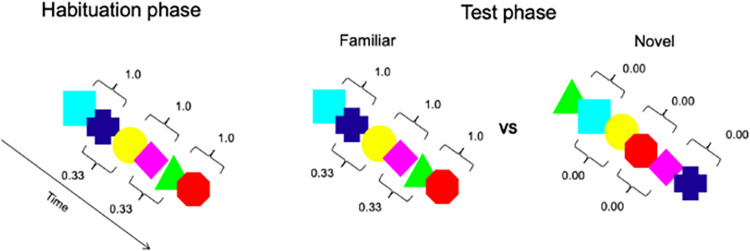
Schematic representation of the visual SL task.

Following Bettoni et al. [30], each infant was tested in an infant-controlled visual habituation task. During habituation, infants viewed six colored shapes (turquoise square, blue cross, yellow circle, pink diamond, green triangle, and red octagon) on a black background presented one at a time for 750 ms in the center of the screen. Each shape loomed along the vertical and horizontal axes from 3 cm to 10 cm. Shapes were presented in a continuous stream without any break or delay between them and were organized into three pairs presented in random order (Fig 1). In this way, the transitional probability between shapes was 1.0 within each pair and .33 between pairs. Habituation trials started with the appearance in the center of the screen of a cartoon animated image associated with varying sounds, which served as an attention-getter. As soon as the infant fixated on the screen, the experimenter turned off the cartoon and started the sequence presentation. Each habituation trial was presented for a minimum of 500 ms and lasted until the infants looked away from the screen for two consecutive seconds. The habituation phase ended when the cumulative looking time on three consecutive trials decreased by 50% of the cumulative looking time for the first three habituation trials [42]. If infants did not meet the habituation criteria, the habituation phase was presented until the infant viewed 21 trials [43]. The 21 trials as a maximum number of trials to habituate was used to maximize the possibility that both HL-ASD and NT infants complete the task [e.g., 44].

In the test phase, infants viewed six test trials in which the familiar (statistically structured) and novel sequences were presented in alternation. Each trial ended when infants looked away from the screen for two consecutive seconds or accumulated a maximum looking time of 60 seconds. The order of presentation (novel first vs. familiar first) was counterbalanced across participants. To be included in the analysis during the test phase, each infant had to complete the 6 test trials and look at the stimuli for at least 1 second in each trial. The novel sequences were characterized by 6 shapes presented in random order with the only constraint that no more than two identical shapes could appear in a row. As a result, the transitional probability between the shapes in the novel sequences was 0. Both habituation and test trials were presented until the infant looked away from the screen for at least 2 consecutive seconds.

Looking time (s) toward the test sequences was used as the dependent variable. For about half of the participants (n = 20), looking times during test trials were coded offline by a second independent observer who was blind to the stimuli presented. Inter-observer agreement between the two observers who coded the data live or from a digital recording, as computed on total fixation times on each of the six test trials, was r = .985 (*p* < .001, Pearson correlation).

The dependent variables for the habituation phase were (1) looking times, (2) number of trials to habituate, and (3) slope of the habituation looking times, which were calculated by fitting linear regression functions to each infant’s online-coded looking time (in seconds) across the habituation trials. The dependent variable for the test phase was the looking times toward familiar and novel sequences.

### Autism Diagnostic Observation Schedule (ADOS-2)

The Autism Diagnostic Observation Schedule– 2^nd^ Edition (ADOS-2) is a semi-structured assessment of communication, social interaction, and restricted/repetitive behaviors for individuals suspected of having ASD [45]. The ADOS-2 includes five modules depending on different developmental ages and language levels. In this study, we used the ADOS Toddler module and the ADOS Toddler Module 1 to evaluate the severity of ASD symptoms at 24 and 36 months. The clinician codes the behaviors exhibited by the child throughout the whole session. In particular, Social Affect (SA) behaviors included: frequency of vocalization directed to others, pointing, gestures, unusual eye contact, facial expressions directed to others, integration of gaze during social overtures, shared enjoyment in interaction, showing, joint attention, and quality of social overtures. Furthermore, Restricted and Repetitive Behaviors (RRB) included: intonation of vocalizations, stereotyped/idiosyncratic use of words or phrases, unusual sensory interest in play material/person, hand and finger and other complex mannerisms, unusual repetitive interests or stereotyped behaviors.

Calibrated Severity Score (CSS) was calculated for each participant using the Total, SA, and RRB scores. The CSS range is 1 to 10 and makes it possible to compare different modules of ADOS, controlling for participants’ age and language levels [46, 47]. Notably, no significant differences were found between the two versions for total SA and RRB subscales (*p*_*s*_ > .56). The descriptive statistics for ADOS measures were: ADOS SA CSS (*M* = 3.63; *SD* = 2.68), ADOS RRB CSS (*M* = 3.75; *SD* = 2.29), and ADOS total CSS (*M* = 3.25; *SD* = 2.86).

## Results

### Habituation phase

In order to compare the performance between the HL-ASD and NT infants during the habituation phase, we run a linear mixed model (LMM, lme4 R package [49] on R software version 4.2.3 [50]), using the default Satterthwaite approximations for degrees of freedom, that should be preferred when dealing with smaller numbers of subjects and items [48]. The looking time in each habituation trial was entered in the LMM as a dependent variable, Group (HL-ASD, NT) as a categorical predictor variable, Habituation Trial as a covariate, and Subjects and Habituation Trial as the random intercept.

The model tested was:

Looking Time ~ Group * Habituation Trial + (1+ Habituation Trial | ID)

**Fig 2:**
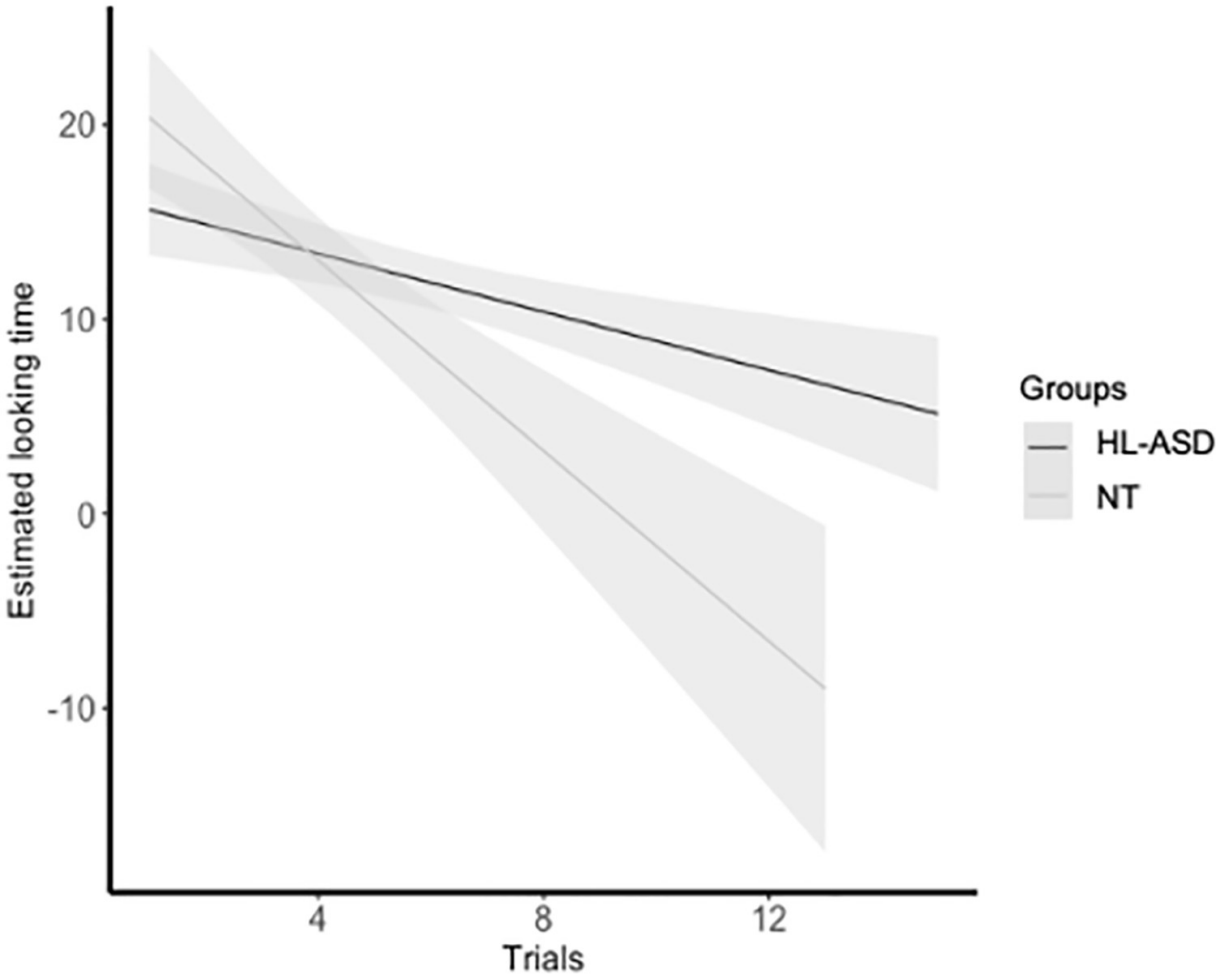
Estimated looking time across the habituation trials for each group.

The analysis revealed a Group x Habituation Trial interaction (*B* = 2.318, *SE* = 0.822, *t* = 2.818, *p* = 0.009), suggesting that the looking time of NT shows a steeper slope compared to the HL-ASD group (see Fig 2). The analysis revealed a tendency to the significance of the factor Habituation Trial (*B* = 1.129, *SE* = 0.546, *t* = 2.069, *p* = 0.052), suggesting that the looking times vary across trials from the first to the last one. Finally, the Group effect did not reach significance (*p* > .102). Since this LMM may not fully account for the distribution of the infant’s looking time (Skewness = 3.940, Kurtosis = 25.295; Kolmogorov-Smirnov test, *p* = .795), we have fitted the model using a Generalized Linear Mixed Model (GLMM, lme4 R package [49]) on raw-looking time in accordance with the gamma distribution of the dependent variable and with transformed-looking time data connected by the log link function [e.g., 51, 52]. The result overlapped with the LMM analysis, showing a significant Group x Habituation Trial interaction (*B* = -0.138, *SE* = 0.041, *t* = 3.325, *p* < .001) and an effect of the Habituation Trial (*B* = -0.129, *SE* = 0.029, *t* = 4.413, *p* < .001), while the Group factor did not provide a significant effect (*p* > .094).

### Test phase

We run a linear mixed model (LMM, *lme4* R package [49] on R software version 4.2.3 [50]), using the default Satterthwaite approximations for degrees of freedom [48]. The looking time in each test trial was entered in the LMM as a dependent variable, Test Trial Type (familiar, novel), Group (HL-ASD, NT), and Test Trial Order (novel, familiar) as a categorical predictor variable, Participant’s Age as covariate and Subjects as random intercept.

The model tested was:

Looking Time ~ Test Trial Type + Test Trial Order + Group + Age in Days + (Test Trial Type: Test Trial Order) + (Test Trial Type: Group) + (Test Trial Order: Group) + (Test Trial Type: Test Trial Order: Group) + (1 | ID)

**Fig 3:**
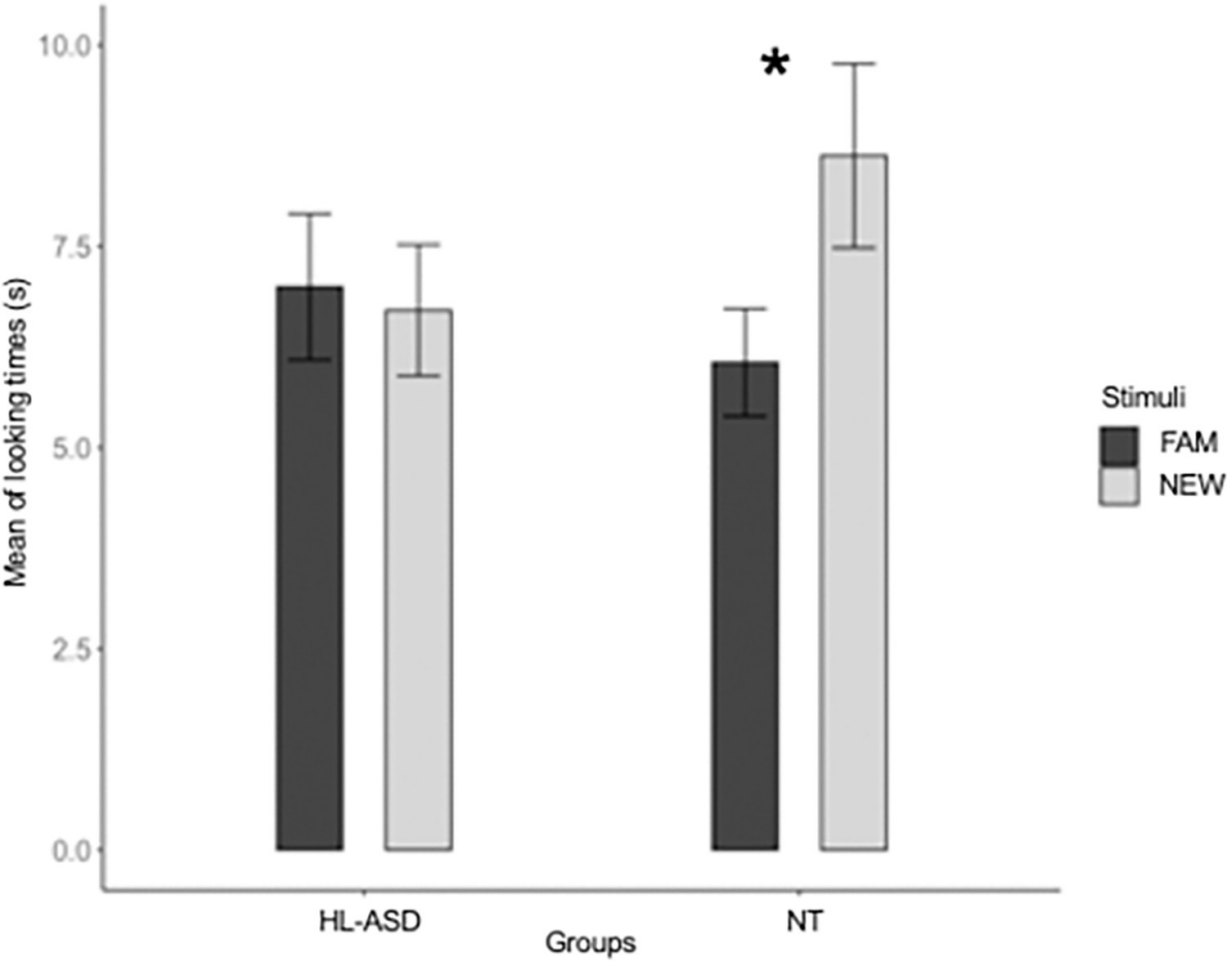
Mean of looking times to the novel and familiar test stimuli in the two groups. *p < .05.

The analysis revealed a Group x Test Trial Type interaction (*B* = 4.494, *SE* = 2.053, *t* = 2.189, *p* = .030). Post-hoc analyses (Bonferroni corrected) revealed that the NT infants looked longer to the novel test sequences (*M* = 8.73 s, *SD* = 3.81) than to the familiar ones (*M* = 6.12 s, *SD* = 3.81; *p* = .030), while HL-ASD infants did not show differences in looking times to the novel (*M* = 6.78 s, *SD* = 3.94) vs. the familiar test stimuli (*M* = 7.32 s, *SD* = 3.98; *p* = 1.00) (Fig 3). No other effects attained significance (*p*_*s*_ > .323). Since the LMM may not fully account for the distribution of the infant’s looking time (Skewness = 2.022, Kurtosis = 8.549; Kolmogorov-Smirnov test, *p* = .582), we have fitted the model using a Generalized Linear Mixed Model (GLMM, *lme4* R package [49]) on raw-looking time in accordance with the gamma distribution of the dependent variable and with transformed-looking time data connected by the log link function [e.g., 51, 52]. The result overlapped with the LMM analysis, showing a significant Group x Test Trial Type interaction (*p* < .038). Specifically, NT infants significantly look longer to the novel stimuli compared to the familiar ones (*p* < .020), whereas no significant difference in the duration of looking times to novel and familiar sequences was observed for HL-ASD (*p* = 1.00).

### Exploratory associations between SL abilities at 6 months and ASD-related traits at 24–36 months

At the preliminary level, we explore the association between visual SL at 6 months and later ASD symptoms at 24 and 36 months. We conducted a linear regression analysis in the HL-ASD sample, including the proportional delta scores obtained from looking times to the novel and familiar test sequences (Novel − Familiar/Novel + Familiar) as the independent variable and the ADOS calibrated severity scores-CSS (ADOS Social Affect-SA CSS, ADOS Restricted/Repetitive Behavior-RRB CSS, and total ADOS CSS scores) as dependent variables, controlling for potential age and gender effects. Regression analysis gives important information about how much the response (ADOS-2) changes for every one-unit increase in proportional delta scores obtained from looking times to the novel and familiar test sequences. Specifically, the proportional delta score was used as a dependent variable in order to account for the individual variabilities in the duration of the infant’s fixation [53]. Since that in-person evaluation was suspended during the COVID-19 pandemic, we do not have follow-up measures for three HL-ASD children. Therefore, the final sample for this second analysis was composed of 16 HL-ASD infants.

**Fig 4:**
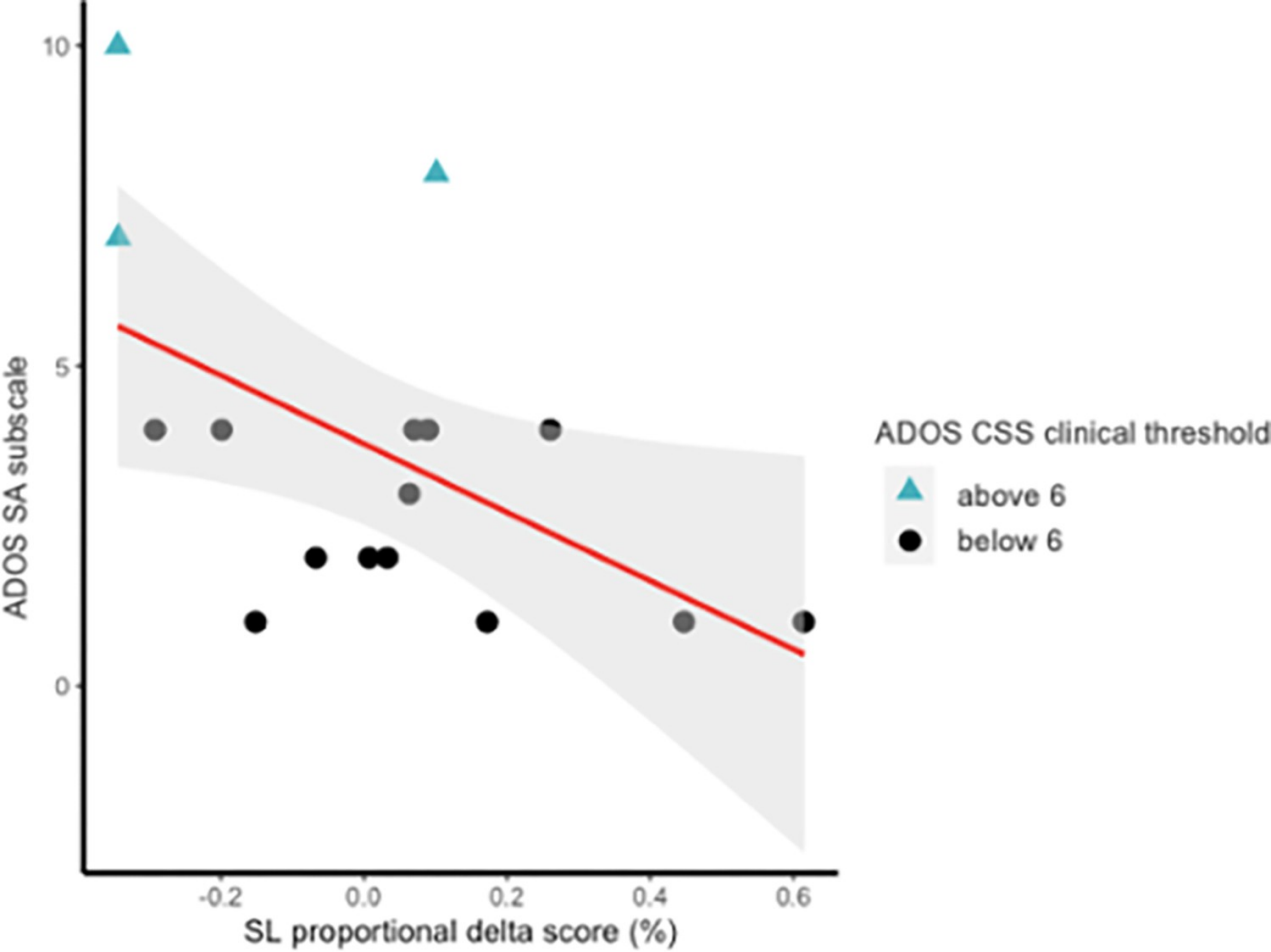
ADOS calibrated severity scores Social Affect subscale assessed at 24–36 months plotted as a function of infants’ SL proportional delta scores (Novel − Familiar/Novel + Familiar) recorded at 6 months.

Results showed a significant association between SL proportional delta scores and ADOS SA CSS (unstandardized *β* = −6.90, *p* = .016; FDR-adjusted *p-value* = .048), as children with higher ASD symptoms at 24–36 months showed lower delta scores in the visual SL task (Fig 4), suggesting the presence of difficulty at discriminating between the random and statistically structured test sequences. Moreover, we found a significant association between SL proportional delta scores and ADOS total CSS scores (unstandardized *β* = −6.46, *p* = .040). However, this effect did not survive when correction for multiple testing was applied (FDR-adjusted *p-value* = .060). We found no significant associations between SL proportional delta scores and ADOS Restricted/Repetitive Behavior-RRB (unstandardized *β* = −1.60, *p* = .541; FDR-adjusted *p-value* = .541).

## Discussion

The present study aimed to investigate the functioning of visual SL in infants at higher likelihood of developing ASD and its predictive value on later ASD-related traits at 24–36 months.

Results show that, after being habituated to a statistically structured visual sequence, 6-month-old NT infants were able to discriminate the habituation sequence from a random sequence. Conversely, the HL-ASD group of infants did not show any discrimination ability, suggesting the presence of a different pattern in extracting the statistical relationships embedded in the visual sequences compared to the NT group. Even though the lack of discrimination on average might lead to hypothesizing a deficit in VSL in HL-ASD, it is important to consider the heterogeneity in performing visual SL tasks, which is often reported to vary greatly in HL-ASD infants [32] and children with ASD [27]. For example, Marin et al. [32] found a different neural response in 3-month-old HL-ASD infants compared to NT infants, indicating a different learning pattern that is associated with nonverbal cognitive skills. Similarly, in our study, the group-level differences in VSL between the HL-ASD and NT infants may reflect differences in learning patterns rather than a deficit in extracting statistical relationships per se. This interpretation is further supported when considering the regression analysis by which HL-ASD infants who showed learning patterns similar to NT infants develop less severe autism symptoms. The difference in the test performance between the two groups was accompanied by a difference in the habituation behavior. Indeed, while the amount of habituation time did not differ between the two groups, HL-ASD infants showed a flatter slope of looking duration compared to NT infants. These results closely resemble previous studies that demonstrated differences in learning visual statistical patterns in HL-ASD infants [32] and in the habituation process in responses to repeated visual stimuli in adults and children with a diagnosis of ASD [54–56]. However, since studies on habituation behavior in individuals with ASD have yielded conflicting results [54], further research is needed to confirm and replicate these results.

In addition to the high heterogeneity in performing visual SL tasks, it may be plausible that HL-ASD infant’s variability in differentiating between the familiar and the novel stimuli in the test phase is related to differences in generalizing statistical regularities learned during the habituation to different contexts. Indeed, at the habituation phase, infants were exposed to a continuous stream of shapes containing unchanging statistical regularities, while at the test, they viewed the familiar structure intermixed with a novel one, as familiar and novel stimuli were presented in alternation. Therefore, HL-ASD infants may perceive familiar structures at the test as novel as those embedded in the real novel sequences (see also [30]).

Alternatively, the different visual patterns shown by the HL-ASD infants between the novel and familiar stimuli at the test may be related to a difference in the deployment of visual attention toward the relevant (statistical) information, as it has been shown that spared attentional resources are needed to compute the statistical relationships in the input stream successfully [57–60]. Thus, statistical information might have captured visual attention efficiently in the NT infants, who were indeed able to use the distributional cues between and within pairs to couple the different shapes into highly predictable units, but not in the HL-ASD group of infants, who might have encoded the statistically structured sequence as a series of an individual object or single chunk. This attentional bias toward the task-irrelevant dimensions of the stimuli, i.e., toward the local features of the item, might have reduced the likelihood of linking the different items across time, preventing the HL-ASD infants from discriminating the structured sequence from the random one at the test. Many studies have shown that siblings of children with ASD exhibit specific characteristics in the orienting of attention [61–63] and that such features might be related to a detail-oriented perception often reported in ASD [61, 64–66]; see also "weak central coherence" by Frith [67]. Both differences in disengaging and shifting visual attention and in focusing attention on local details might have prevented Statistical Learning in HL-ASD infants. However, our study does not allow us to test whether variability in visual SL is a cause or a consequence of attentional characteristics. Further studies are needed to investigate the role of the attentional processes in visual SL skills in typical and atypical development.

Finally, the prediction coding account of ASD [24–26] posits that individuals with ASD might downweigh prior experiences in favor of recent ones, and this tendency could result in an elevated perception of novelty, leading to an increased “hyperplasticity” in learning based on the most recent input. An overweighting of the most recent input rather than the cumulative aggregation of instances [24, 25] might, in turn, prompt individuals with ASD to update their predictions continually and unnecessarily about the world. This characteristic poses challenges in perceiving statistical relationships, particularly in environments with high variability, relative to when it remains relatively stable. In our study, the visual input contained various probabilities regarding the occurrence of the shape in the sequence: a high probability (deterministic), defined by the onset of the second shape in the pair, and a low probability (probabilistic), defined by the onset of the first shape in the pair. Contrary to the NT group, HL-ASD infants might have increased neural resources toward probabilistic rather than deterministic information [32], which might prompt a continuous update of their predictive model. This constant updating might not allow them to build a robust representation of the statistical structure, including the deterministic vs. probabilistic information, thereby challenging their ability to discriminate between familiar and novel structures at test. Therefore, it is plausible to hypothesize that differences between NT and HL-ASD infants in discriminating the novel test stimuli from the familiar ones might be due to an improper weighting of prediction errors rather than to difficulties in statistical learning per se. Future studies should investigate different distributions of the input to HL-ASD infants to provide further evidence on the prediction coding account of ASD.

To the best of our knowledge, our findings are the first to show different visual statistical learning patterns in HL-ASD infants at the behavioral level, extending prior work demonstrating different patterns of brain activity in processing both auditory and visual statistical dependencies in siblings of children with ASD [32, 68]. These results are also consistent with our previous study [43], showing that infants whose parents had higher autistic traits exhibited visual SL dysfunctions compared to infants whose parents had lower autistic traits. The convergence between the present study results and our previous study (i.e., [30]) supports the hypothesis that biological siblings of children with ASD and individuals with broader autism phenotypes belonging to the general population shared similar differences in statistical learning patterns in early visual SL [69].

Finally, the regression analysis showed that the different ability to discriminate between sequences containing highly predictable and unpredictable statistical relationships at 6 months of age was associated with increased social communication and interaction deficits, as measured by ADOS Social Affect at 24–36 months. These results further support the idea that visual SL plays a critical role in social and communicative skills in ASD [17, 30, 32, see 66]. Indeed, the ability to learn regularities is important for segmenting the continuous flux of information, helping infants to find words in the speech and action streams [21, 22]. Infants might use their knowledge about the relationship among action units to generate an expectation about what action is likely to follow the action they just viewed [e.g., 25, 70]. Therefore, learning such pairings between antecedent and consequence events may provide the foundation for forming predictions about upcoming social experiences [12, 20–22]. For example, a recent study on the role of infants’ visual SL in action processing found motor cortex activation during anticipation of upcoming actions that were statistically determined [71]. Notably, research investigating the role of SL in understanding motion in naturalistic contexts have shown that infants extracted those units that tend to be closely related to the intention and goals of the actor. Specifically, infants segment the continuous flow of actions into units based on the completion or initiation of the actor’s intentions [21]. These results further suggest that the infant’s brain catches the statistical relationship in the environment and uses such information as a temporal cue to create expectations about social events, such as the agent’s intention [21]. Therefore, differences in visual SL may have cascading effects on the ability to learn behaviorally relevant structures, which might preclude the acquisition of skills needed to generate expectations about upcoming socially relevant information and adapt to the social environment (e.g., [16]).

Overall, our findings suggest a link between early visual SL variabilities and later social and communicative skills that characterize ASD-related traits. Beyond the specific contribution of visual SL in explaining autistic features, research focusing on autism in infancy needs to examine interactions among several general and specific neurocognitive domains to understand how different factors could explain individual differences and heterogeneity in ASD [72, 73].

It is worth noting that our study has some limitations that should be mentioned. We did not assess the outcome in the NT sample, and future studies should be devoted to extending the association between SL abilities in preverbal infants and later social/communicative skills in neurotypical populations. Additionally, even if the two groups did not differ in the cognitive abilities at 7 months, we could not exclude that group differences might not be attributed to a general development delay [32]. The investigation of the interplay among diverse neurocognitive domains such as attention, cognition, and social skills can provide a further understanding of how these elements integrate and mutually shape one another during early development. Finally, only three infants later showed ASD-related outcomes, with one infant displaying a novelty bias similar to that of neurotypical (NT) infants. Thus, the results should be interpreted with caution due to the small sample size and a larger HL-ASD sample is needed to extend further the results about the nuance of SL patterns in autism.

In conclusion, the current findings add to a growing body of literature supporting the presence of early behavioral markers in siblings of children with ASD before the disorder fully expresses itself. The characterization of early markers will guide the detection of the most vulnerable infants who might benefit from early intervention.
